# Injectable long acting antiretroviral for HIV treatment and prevention: perspectives of potential users

**DOI:** 10.1186/s12879-023-08071-9

**Published:** 2023-02-17

**Authors:** Laurence Slama, Raphael Porcher, Françoise Linard, Catherine Chakvetadze, Agnès Cros, Séverine Carillon, Lucille Gallardo, Jean-Paul Viard, Jean-Michel Molina

**Affiliations:** 1 Department of Infectious Diseases, Hôtel Dieu Hospital, APHP, 1 Place du Parvis Notre-Dame, 75004 Paris, France; 2 Université Paris Cité and Université Sorbonne Paris Nord, Inserm, INRAE, Center for Research in Epidemiology and StatisticS (CRESS), 75004 Paris, France; 3 Centre d’Épidémiologie Clinique, AP-HP, Hôtel-Dieu, 75004 Paris, France; 4 Department of Infectious Diseases, Tenon Hospital, APHP, 75020 Paris, France; 5 Departement of Infectious Diseases, Groupe Hospitalier Sud Île de France, Melun, France; 6 COREVIH Île de France Sud, Créteil, France; 7 CEPED, IRD, Paris Cité University, Paris, France; 8 Mesopolhis, AMU/IEP, Aix-en-Provence, France; 9 Paris Cité University, Paris, France; 10 Departement of Infectious Diseases, Saint Louis and Lariboisière Hospitals, APHP, Paris, France; 11 INSERM U944, Paris, France

**Keywords:** Long-acting cART, HIV treatment, Quantitative study, HIV prevention, PrEP

## Abstract

**Background:**

The use of long acting injectable (LAA) antiretroviral drugs may be an alternative option for HIV treatment and prevention. Our study focused on patient perspectives to understand which individuals, among people with HIV (PWH) and pre-exposure prophylaxis (PrEP) users, would constitute the preferential target for such treatments in terms of expectations, tolerability, adherence and quality of life.

**Methods:**

The study consisted in one self-administrated questionnaire. Data collected included lifestyle issues, medical history, perceived benefits and inconveniences of LAA. Groups were compared using Wilcoxon rank tests or Fisher’s exact test.

**Results:**

In 2018, 100 PWH and 100 PrEP users were enrolled. Overall, 74% of PWH and 89% of PrEP users expressed interest for LAA with a significantly higher rate for PrEP users (p = 0.001). No characteristics were associated with acceptance of LAA in both groups in term of demographics, lifestyle or comorbidities.

**Conclusion:**

PWH and PrEP users expressed a high level of interest in LAA, since a large majority seems to be in favor of this new approach. Further studies should be conducted to better characterize targeted individuals.

**Supplementary Information:**

The online version contains supplementary material available at 10.1186/s12879-023-08071-9.

## Background

Oral preexposure prophylaxis (PrEP) and combined antiretroviral therapy (cART) are cornerstones to overcome the HIV epidemic [1, 2]. However, despite their high effectiveness, their better tolerability and convenience, they have limitations that may affect quality of life and adherence [3]: tiredness of daily long-term treatment, difficulty experienced by some individuals in swallowing pills, potential drug interactions with comedications such as proton pump inhibitors [4].

To overcome these drawbacks, developing alternative forms of cART such as long acting injectable antiretroviral (LAA) drugs is likely to be needed [5].

The use of long acting treatments has already been studied in several fields of medicine such as psychiatry, for the treatment of psychosis, contraception, with implants [6], or osteoporosis [7], with major challenges. As an example, long acting neuroleptics have been developed since 1970 to prevent non adherence and relapse: even if they have demonstrated their effectiveness [8, 9], the therapeutic link remains essential for a sustainable success.

Recent publications indicate that the use of LAA may be an alternative option in the field of HIV treatment and prevention [10, 11]. Expectations are multiple including less frequent dosing, less drug-drug interactions, less adverse events. Moreover, potential users would expect more protection of health privacy, avoiding pill fatigue and perhaps improving adherence issues [10]. Therefore, it seems extremely important to fully understand the expectations and risks of long-acting treatment implementation for both HIV prevention and treatment [12].

A first long acting regimen was recently approved [1, 4] consisting of an association of an integrase inhibitor (cabotegravir-long acting, CAB-LA) and a non-nucleoside reverse transcriptase (NNRTI) (rilpivirine-long acting, RPV-LA) [13, 14] for HIV-infected adults with viral suppression (HIV RNA < 50 copies/ml), with no suspected resistance to either cabotegravir or rilpivirine. This approval was based on the ATLAS 2M study which demonstrated the non-inferiority of CAB-LA + RPV-LA combined regimen dosed every 8 weeks compared to CAB-LA + RPV-LA dosed every 4 weeks [15]. Regarding HIV prevention, two major trials demonstrated a clear superiority of CAB-LA alone compared to the standard one-pill-once-a-day tenofovir disoproxil fumarate/ emtricitabine (TDF/FTC) regimen in cisgender men, cisgender women and transgender women [16, 17]. Overall, this new mode of administration could provide a new option for HIV-infected patients and be an effective strategy in preventing HIV infection.

However, many questions remain unresolved. How to identify potential users? By whom, where and how will treatments be injected? How to manage injections every two months in already busy healthcare facilities? How to organize injection visits within the appropriate time window (± 7 days) to avoid adherence issues and selection of resistance mutations? And finally, what are patients’ expectations regarding this new mode of administration?

Switching from a standard one pill-once a day regimen to an injection every other month would set a new paradigm, susceptible to shake people with HIV (PWH), PreP users’ and health care providers’ habits.

The purpose of our study was to focus on patient perspectives and to understand which individuals, among PWH and PrEP users, would constitute the preferential target for such treatments in terms of expectations but also tolerability, adherence and improvement in quality of life.

## Methods

The ANRS CLAPT project consisted in two complementary quantitative and qualitative studies among PWH and PrEP users. The qualitative study have already been published elsewhere [18–20]. The quantitative study consisted in one self-administered questionnaire among PWH and PrEP users in three Hospitals in France (Hôtel-Dieu University Hospital, Paris; Saint-Louis University Hospital, Paris, and Melun Hospital, Melun). Data collected from participants included lifestyle issues, medical history, treatment experience, perceived benefits and inconveniences of LAA. Not fully aware of the future approved indication for LAA at the time of the study, no particular drug was mentioned. The objective of this research was rather to describe individuals’ perceptions in terms of a new route of drug administration and to assess its acceptability. However, for ethical reasons and to minimize potential disappointment for future users, all PWH had to be ART-treated with HIV RNA < 50 copies/ml, and PrEP users on TDF/FTC had to have at least a 6-month follow-up to be included**.** One out of two PWH or PrEP users, meeting the inclusion criteria were randomly included during each consultation. For logistical reasons, no more than three patients were included per consultation session. According to the number of PWH followed by each center, it has been decided to include 40 PWH in Saint-Louis and Hôtel-Dieu and 20 PWH in Melun centers, respectively.

The study obtained the approval of the Committee for the Protection of Persons on May 30, 2018 (reference: 2018-A01527-48). Each participant gave written informed consent in order to respond to the self-administrated questionnaire.

A team of doctors and anthropologists built questionnaires for both PWH and PrEP users. Detailed questionnaires, developed for this study, were in French language and were secondary translated and added as supplementary files (Additional files 1, 2). However, all survey were responded in French.

The number of refusals has not been recorded but represents less than 5% of the studied population, mainly linked to the unavailability of individuals to stay longer after the consultation session.

Gender, age, country of birth were collected for all respondents as well as family situation (in a relationship/single, with or without children), occupation, travels frequency (less/more than twice a year, never), lifestyle (alcohol consumption, tobacco use: yes/no/former, inhaled or injected illicit drugs: yes/no), self-declared comorbidities (high blood pressure, treated high blood pressure, diabetes, treated diabetes, dyslipidemia, treated dyslipidemia, depression, treated depression: yes/no) and contraception use (yes/no).

Regarding PWH, collected items were: date of HIV diagnosis, route of HIV transmission, ART exposure duration, tolerance and adherence to current ART regimen, intake frequency and associated treatments as well as each individual's perception of their ART history experience. At the same time doctors collected for each PWH the CDC stage, CD4 cell count nadir, current CD4 count and viral load as well as the ART history classified as two nucleoside reverse transcriptase inhibitors plus either a boosted protease inhibitor, a non-nucleoside reverse transcriptase inhibitor, an integrase inhibitor or other combinations (Additional file 3).

PrEP users were specifically asked about number of sexual partners in the past 3 months, length of PrEP exposure as well as tolerance and adherence to PrEP.

For both PWH and PrEP users, specific questions were asked concerning LAA that offered predefined answers: perception of the advantages and disadvantages of this new mode of administration, acceptability of the frequency of injections, acceptability of the idea to come to the medical care unit more often and the desire to switch from current therapies to LAA.

Groups were compared using Wilcoxon ranks tests for continuous variables or Fisher’s exact tests for proportions. Participants characteristics associated with LAA acceptability were analyzed separately for PWH and PrEP users. Given the high number of tests, p-values < 0.005 were considered as indicating statistical significance.

## Results

Between October and December 2018, 200 respondents (100 PWH and 100 PrEP users) were enrolled, with a majority of men (76 and 100%, respectively). Men who have sex with men (MSM) represented 50% and 100% of the PWH and PrEP users, respectively. A description of the participants is provided in Table 1. Unsurprisingly, PWH were older (median age (IQR): 50 years (42–56)) compared to PrEP users (39 years (31–45), p < 0.0001), with a higher rate of reported hypertension (25 vs. 8% for PWH and PrEP users, respectively, p = 0.002) and dyslipidemia (33% vs. 2%, p < 0.0001). Illicit drug consumption was more frequent among PrEP users compared to PWH (48% and 20% among PrEP and PWH, respectively, p < 0.0001) whereas 22% were current smokers in both groups (p = ns).

**Table 1:** Demographic characteristics of PWH and PrEP users

Variable	HIV N = 100	Prep N = 100	p
Median age (IQR), year	50 (42–56)	39 (31–45)	< 0.0001
Men, n	76	100	< 0.0001
Sexual orientation, MSM, n	50	100	< 0.0001
Country of birth, France, n	63	79	0.019
Family life, single n	59	81	0.001
Has children, no	42	4	< 0.0001
Worker, no. (%)	71	90	0.001
Travels, no. (%)			< 0.0001
1–2/year	48	23	
> 2/year	34	75	
Never	17	2	
Smoking status, current n	22	23	> 0.99
Alcohol consumption, n	79	87	0.19
Illicit drugs consumption, n	28	56	< 0.0001
Hypertension, n	25	8	0.002
Dyslipidemia, n	33	2	< 0.0001
Diabetes mellitus, n	8	4	0.37
Depression, n	15	17	0.85
Antidepressant, n	9	5	0.41
Past experience with injectable, n	35	56	0.004
Partner aware of HIV or prep user status, n	76	91	0.007
Family aware of HIV or prep user status, n	48	24	0.0007
Friends aware of HIV or prep user status, n	47	74	0.0002
Hepatitis B virus coinfection, n	11	–	
Hepatitis C virus coinfection, n	11	–	
HIV exposure, years, mean, n	15	–	
ART exposure > 10 years, n	58	–	
Stade CDC, C, n	15	–	
CD4 nadir (/mm3), mean	325	–	
Current CD4 cell count (/mm3), mean	748	–	
Viral load HIV-RNA < 50 copies/ml	100	–	
Actual ART regimen OAD, n	95	–	
Actual ART regimen STD, n	65	–	
2NRTI + NNRTI, n	30	–	
2 NRTI + IP/r, n	9		
2 NRTI + II, n	52	–	
Other combination, n	9	–	

Compared to PrEP users, PWH were more likely to be involved in a relationship (41% vs. 19% for PWH and PrEP users, respectively, p = 0.001), to have children (42% vs. 4%, p < 0.0001) but were less likely to travel than PrEP users (p < 0.0001). Concerning their entourage, almost half of PWH had shared their HIV status their family (48% vs 24% for PWH and PrEP users, p = 0.0007), while PrEP users had shared their PrEP users status with friends (74% vs 47% for PrEP users and PWH, p = 0.0002).

Looking at HIV parameters in the PWH group, participants had been living with HIV for a mean time of 15 years and 58% of them had been ART-treated for at least 10 years. A large majority (93%) received a once-a-day ART regimen and 65% a single tablet daily (STD). All participants were well controlled (HIV RNA < 50 copies/ml) with a mean current CD4 cell count at 748/mm^3^.

### LAA acceptability in PWH and PrEP users (Table 2)

**Table 2:** Acceptability questionnaires in PWH and PrEP users

Variable	HIV N = 100	Prep N = 100	*P*
Would accept LAA every other month, n (%)	74 (74)	89 (89)	0.001
Perceived advantages			
Stop taking cART^a^-treatment every day, n (%)	73 (73)	73 (73)	> 0.99
Being certain of efficacy for a period of time, n (%)	42 (42)	51 (51)	0.26
Being certain not to forget cART-treatment, n (%)	39 (39)	68 (68)	< 0.0001
Hiding that I take cART-treatment, n (%)	0 (0)	7 (7)	0.014
No need to think of treatment every day, n (%)	28 (28)	62 (62)	< 0.0001
Forget the disease, n (%)	19 (19)	–	–
Other, n (%)	11 (11)	0 (0)	0.0007
None, n (%)	11 (11)	0 (0)	0.0007
Perceived drawbacks			
Loss of freedom, n (%)	6 (6)	30 (30)	< 0.0001
Fear of adverse effects, n (%)	29 (29)	47 (47)	0.013
Fear of injections, n (%)	14 (14)	19 (19)	0.45
Fear of being treated as a Guinea pig, n (%)	17 (17)	23 (23)	0.38
It would not change anything, n (%)	9 (9)	8 (8)	> 0.99
Already taking other treatments, n (%)	7 (7)	1 (1)	0.065
Too much a constraint, n (%)	21 (21)	0 (0)	< 0.0001
Other, n (%)	6 (6)	0 (0)	0.029
None, n (%)	29 (29)	22 (22)	0.33
What do you feel about coming every other month?			
Beneficial because more follow-up, n (%)	23 (23)	28 (28)	0.52
I don't like the idea of going more often to the hospital, n (%)	34 (34)	21 (21)	0.057
I don't care, n (%)	42 (42)	53 (53)	0.16
About an injectable treatment?			
Do not wish to change my treatment, n (%)	23 (23)	12 (12)	0.062
Accepts both injections and hospital visits, n (%)	35 (35)	58 (58)	0.002
Accepts injections but not hospital visits, n (%)	29 (29)	21 (21)	0.25
Accepts injections but fears adverse effects, n (%)	18 (18)	19 (19)	> 0.99
Would like to switch to injections at specific times, n (%)	27 (27)	29 (29)	0.87
Would you accept participating to a clinical trial?			
Would not participate, n (%)	28 (28)	31 (31)	0.76
Would accept injections at the hospital, n (%)	34 (34)	36 (36)	0.88
Could accept injections if self-administered, n (%)	19 (19)	7 (7)	0.019

^a^ART: combined antiretroviral therapies

Overall, 74% of PWH and 89% of PrEP users expressed interest for every other month LAA with a significantly higher rate for PrEP users (p = 0.001).

Perceived advantages and drawbacks are reported in Table 2. Perceived benefits were mostly expressed by PrEP users and consisted in being sure not to miss daily medication (68% and 39% for PrEP users and PWH, respectively, p < 0.0001) and to avoid the burden of thinking about taking medication every day (62% and 28% for PrEP users and PWH, respectively, p < 0.0001). Regarding PWH, we notice that 11% of them did not perceive any benefit for this type of administration, which never was the case among PrEP users (p = 0.0007). Unexpectedly, even if the PrEP users were those who had most perceived expected advantages, they were also more frequently underlining disadvantages concerning this new approach: fear of losing their freedom (30% and 6% for PrEP users and PWH, respectively, p < 0.0001) and apprehension of potential side effects (47% and 29%, for PrEP users and PWH, respectively, p = 0.013). In addition, among PWH, we noticed again a greater fear of too much a constraint linked to the new mode of administration compared to the PrEP users group (21% and 0% for PWH and PrEP users, respectively, p < 0.0001). Overall, we find a higher proportion of PrEP users wiiling to accept both injections and hospital visits compared to PWH (58% and 35%, for PrEP users and PWH, respectively, p = 0.002).

### Association between participant characteristics and LAA acceptability

The potential association of the different participant characteristics and acceptance is displayed on Tables 3 for PWH and Table 4 for PrEP users. No characteristics seemed to be associated with acceptance of LAA either in the PWH and the PrEP users groups in term of demographics, lifestyle, comorbidities or HIV parameters in the HIV group, including PWH under a single tablet regimen (Additional file 4: Table S1). Similarly, no statistical difference was found between PrEP users who would accept and those who would not accept injections. It should be noted that there is an imbalance, since the majority in both groups would accept injections (74% and 89% in the PWH and PreP users, respectively).

**Table 3:** Patients characteristics associated with acceptance among PWH (N = 100)

Variable	Would accept injections (N = 74)	Would not accept injections (N = 26)	P
Median age (IQR), years	50 (40–56)	52 (47–59)	0.18
Sex, n (%)	59 (80)	17 (65)	0.18
Mode of transmission, n (%)			0.072
MSM	41 (55)	9 (35)	
Heterosexual	21 (28)	12 (46)	
Other^a^	12 (16)	5 (19)	
Family life: single, n (%)	46 (62)	13 (50)	0.35
Has children, n (%)	29 (39)	13 (50)	0.36
Current worker, n (%)	56 (76)	15 (58)	0.13
Travels, n (%)			0.40
1–2/year	34 (47)	14 (54)	
> 2/year	24 (33)	10 (38)	
Never	15 (21)	2 (8)	
Current smokers, n (%)	15 (20)	7 (27)	0.58
Alcohol consumption: yes, n (%)	60 (81)	19 (73)	0.41
Drugs consumption: yes n (%)	16 (22)	4 (15)	0.58
IV drugs consumption: yes n (%)	7 (9)	1 (4)	0.68
CDC stage, n (%)			0.72
C	11 (15)	4 (15)	
Antiviral treatment duration, n (%)			0.24^b^
< 1 year	5 (7)	0 (0)	
1–5 year	17 (23)	4 (15)	
5–10 year	10 (14)	6 (23)	
> 10 year	42 (57)	16 (62)	
Antiviral treatment, n (%)			0.67
Once a day	68 (92)	25 (96)	
STR, n (%)	47 (64)	18 (69)	0.64
Median CD4 (IQR), cells/µl	746 (521–1002)	705 (528–805)	0.56
Median CD4 nadir (IQR), cells/µl	338 (185–446)	292 (150–439)	0.34
Hypertension, n (%)	18 (24)	7 (27)	0.80
Diabetes, n (%)	6 (8)	2 (8)	> 0.99
Hypercholesterolemia, n (%)	20 (27)	13 (50)	0.051
Psychiatric disorder, n (%)	13 (18)	2 (8)	0.34
Antidepressant, n (%)	8 (11)	1 (4)	0.44
Taking any non-HIV treatment, n (%)	34 (46)	11 (42)	0.82
Partner aware of treatment, n (%)	59 (80)	17 (65)	0.18
Family aware of treatment, n (%)	37 (50)	11 (42)	0.65
Friends aware of treatment, n (%)	39 (53)	8 (31)	0.069
Colleagues aware of treatment, n (%)	11 (15)	2 (8)	0.50
Never experienced AEs, n (%)	41 (55)	20 (80)	0.034
Never forgets treatment, n (%)	48 (65)	17 (65)	> 0.99

^a^Others: mother to child, UIVD, unknown

^b^Trend test

*P*-values were obtained by Wilcoxon or Fisher’s exact tests

**Table 4:** Patients characteristics associated with acceptance in Prep users (N = 100)

Variable	Would accept injections^a^ (N = 89)	Would not accept injections (N = 11)	P
Median age (IQR), years	38 (31–45)	41 (37–46)	0.21
Family life: single, n (%)	71 (80)	10 (91)	0.69
Has children, n (%)	4 (4)	0 (0)	> 0.99
Worker, n (%)	80 (90)	10 (91)	> 0.99
Travels, n (%)			0.78
1–2/year	20 (22)	3 (27)	
> 2/year	67 (75)	8 (73)	
Never	2 (2)	0 (0)	
Current smokers, n (%)	22 (25)	1 (9)	0.45
Alcohol consumption: yes, n (%)	78 (88)	9 (82)	0.63
Drugs consumption: yes n (%)	44 (49)	4 (36)	0.53
IV drugs consumption, n (%)	8 (9)	0 (0)	> 0.99
Associative implication, n (%)	4 (4)	0 (0)	> 0.99
Hypertension, n (%)	7 (8)	1 (9)	> 0.99
Treated hypertension, n (%)	4 (4)	1 (9)	0.45
Diabetes, n (%)	4 (4)	0 (0)	> 0.99
Treated diabetes, no. (%)	2 (2)	0 (0)	> 0.99
Hypercholesterolemia, n (%)	1 (1)	1 (9)	0.21
Treated hypercholesterolemia, no. (%)	1 (1)	1 (9)	0.21
Psychiatric disorder, n (%)	15 (17)	2 (18)	> 0.99
Antidepressant, n (%)	4 (4)	1 (9)	0.45
Other disease/condition, no. (%)	12 (13)	3 (27)	0.36
Taking any non-PrEP treatment, n (%)	10 (11)	3 (27)	0.15
Experience with injectable treatment, n (%)	50 (56)	6 (55)	> 0.99
Partner aware of treatment, n (%)	80 (90)	11 (100)	0.59
Family aware of treatment, n (%)	21 (24)	3 (27)	0.72
Friends aware of treatment, n (%)	64 (72)	10 (91)	0.28
Never experienced AEs, n (%)	53 (60)	5 (45)	0.52
Never forgets treatment, n (%)	61 (69)	8 (73)	> 0.99
Median no. partners in the last 3 months (IQR)	12.0 (6–21)	10.0 (5–17)	0.43

^a^*P*-values were obtained by Wilcoxon or Fisher’s exact tests

## Discussion

This quantitative study using a survey on the perception of LAA by PWH and PrEP users yield important data on perceived advantages and drawbacks of this new mode of administration.

First, our study participants expressed a high level of interest in this new mode of administration since a large majority (74% of PWH and 89% of PrEP users) seems to be in favor of this new approach. Our results are consistent with published data [21] mostly conducted by pharmaceutical companies. However, respecting therapeutics windows to avoid any failure of the strategy will be crucial. Unfortunately, we are not in a position to give individuals’ feedback since this question was not addressed in our survey.

Regarding acceptability by PWHs, it appears that a large majority was attracted by this new method even if 92% of our studied population was well controlled (HIV-RNA < 50 copies/ml) with a daily ART (95%) or even a single tablet regimen (64%). Despite the convenience of current cART and similarly to the ATLAS study, which reported a stronger predisposition for LAA in PWH having an extensive experience of daily oral therapies over many years, the high level of acceptability in our PWH study population could be explained by the long duration of both HIV exposure and cART. Moreover, we noticed that all PWH had not shared their HIV status with their entourage suggesting that individuals still hide their HIV status easily revealed through the presence of ART pills at home. This fear of stigmatization [22] could be partly resolved by LAA injections and may also explain this high level of expectations.

Regarding PrEP users, the level of acceptability was high since 89% of our studied population was attracted by LAA. Moreover, it appears that there was a greater advantage expressed by PrEP users compared to PWH, particularly on the risk of missing daily treatments. Indeed, this concern could be easily linked to reported results from PrEP clinical trials, showing a strong correlation between efficacy and drug adherence [23, 24]. In addition, we observed a greater consumption of illicit drugs among PrEP users, known to cause forgetfulness, exposing individuals at a higher risk of HIV acquisition due to adherence issues [25].

Unlike PrEP users, 11% of PWH did not see any benefit in this new mode of administration. This finding could be explained by the higher rate of reported metabolic disorders and comorbidities such as high blood pressure or dyslipidemia among PWH. In line with data from European cohorts [26], these results suggest that, in addition to ART, a large part of PWH, unlike PrEP users, have comedications and therefore cannot avoid daily treatments.

Comparing disadvantages expressed in both groups, PreP users seems more concerned by the “loss of freedom” and “the fear of adverse events” even if a high proportion of them (58%) would accept injections and hospital visits. Regarding PWH, LAA seems to be linked with “too much a constraint” for 21% of them in line with the 11% who do not perceive any benefit. LAA implementation do not represent “too much a constraint” for PrEP users (0% vs 21% for PrEP users and PWH, respectively, p < 0.0001). Overall, it is clear there is more reluctance among PWH that PrEP users even if PrEP users are those who expressed a greater rate of perceived drawbacks.

There was no association between individuals’ characteristics and LAA acceptance for PWH or PrEP users, probably because of the small sample size. In addition, a large majority of PWH and PrEP users being in favor of this new mode of administration, it was not possible to highlight the characteristics of those who would accept compared to those who would not accept LAA. However, there seemed to be trends: individuals accepting LAA would rather be single and younger and those not accepting LAA would be older, involved in a relationship and with associated comorbidities. Naturally, these are only hypotheses that deserve to be verified in larger studies.

Our study has several limitations in addition to those already mentioned. First, even if vulnerable populations were not excluded from our analysis, we were unable to highlight any specificity given the small number of vulnerable persons included. Therefore, our results may not be applicable to women, under-represented in this study, as they may have others health concerns. In the same way, the minorities in France (young people, migrants) have not been studied even though they represent key populations [27]. Specific studies on these populations will have to be done and taken into account to understand the challenges of access to care, HIV diagnosis and treatment in the setting of precariousness and language misunderstanding barriers [28].

Even if LAA could improve treatment adherence, our study does not allow us to anticipate patient’s behaviors in a real-life setting. Experiences with neuroleptic in the field of psychosis showed that patients may perceive this new mode of administration as intrusive, with a loss of autonomy, limiting the freedom to stop drugs [8].

Finally our questionnaire made the assumption of health care delivery in the hospital setting and we were not able to discuss any implementation care (sms reminders, coupling injections with doctor’s consultation, etc.). Since the adherence to injected medications against osteoporosis in menopausal women shows an erosion after few months, a reflection is required to avoid this pitfall [29].

## Conclusion

Overall, PWH and PrEP users expressed a high level of interest in LAA, since a large majority seems to be in favor of this new approach. However, at the time of LAA implementation, it will be a challenge for healthcare professionals to think carefully about its positioning for HIV treatment and prevention. Further studies should be conducted to better characterize individuals who would be the best candidates for these treatments.

## Supplementary Information

**Additional file 1.** « Long acting injectable treatment acceptability » PWH survey: A survey was built in French by a team of doctors and anthropologists for PWH and translate secondarily for publication purpose. Each PWH participant gave written informed consent in order to respond to the self-administrated questionnaire. Data collected included demographic parameters, lifestyle, habits, medical history, HIV history, experiences with medication in particular with ART treatment. Perceived advantages and disadvantages of LAA

**Additional file 2.** « Long acting injectable treatment acceptability» PrEP users survey: A survey was built in French by a team of doctors and anthropologists for PrEP users and translate secondarily for publication purpose. Each PrEP user participant gave written informed consent in order to respond to the self-administrated questionnaire. Data collected included demographic parameters, lifestyle, habits, medical history, PrEP history, experiences with medication in particular with PrEP. Perceived advantages and disadvantages of LAA for PrEP.

**Additional file 3.** « Long acting injectable treatment acceptability» Collected data for PWH by doctors: During the study, data were collected for each participant responding to the self-questionnaire by doctors in the medical file including demographic parameters, habitus, history of HIV (immuno-virologic parameters, ART treatment history, HIV-exposure, ART exposure), comorbidities (HBV, HCV, diabetes, hypertension, dyslipidemia).

**Additional file 4. Table S1**: Patients characteristics associated with acceptance among PWH currently under single tablet regimen (STR), (N=65): A supplementary analyze was done among PWH treated by STR to compare PWH who would accept to PWH who not accept injections.

## Data Availability

The datasets used and/or analysed during the current study are available in the Dr. Porcher repository (raphael.porcher@aphp.fr).

## Abbreviations

CAB-LA: Cabotegravir long acting
cART: Combined antiretroviral therapy
LAA: Long acting injectable antiretroviral drugs
MSM: Men who have sex with men
NNRTI: Non-nucleoside reverse transcriptase inhibitor
PWH: People with HIV
PrEP: Pre-exposure prophylaxis
RPV-LA: Rilpivirine-long acting
TDF/FTC: Tenofovir disoproxil fumarate/emtricitabine
