# Supplementary material for: Injectable long acting antiretroviral for HIV treatment and prevention: perspectives of potential users
Source: BMC Infect Dis. 2023 Feb 17;23:98. doi: 10.1186/s12879-023-08071-9 (PMC9936705; doi:10.1186/s12879-023-08071-9)
Supplement: Supplementary file 1 — Additional file 1. « Long acting injectable treatment acceptability » PWH survey: A survey was built in French by a team of doctors and anthropologists for PWH and translate secondarily for publication purpose. Each PWH participant gave written informed consent in order to respond to the self-administrated questionnaire. Data collected included demographic parameters, lifestyle, habits, medical history, HIV history, experiences with medication in particular with ART treatment. Perceived advantages and disadvantages of LAA [file 12879_2023_8071_MOESM1_ESM.docx]

**Additional file 1: « Long acting injectable treatment acceptability »**

**PWH survey**

**Notice :**

We are carrying out a survey on HIV treatment by injection and would like to hear your opinion.

As you know, the HIV/AIDS epidemic is a public health problem. If we want to improve the management of this disease and in particular antiretroviral treatments, it is important to collect as much information as possible from concerned individuals. It is therefore very important that your point of view is accurately taken into account.

For this, we would like you to answer an anonymous questionnaire lasting about 10 minutes. Thank you very much for your participation in this research and for the time you are willing to devote to it. Your answers will be a very valuable contribution to the research.

For most questions, please give the answer closest to what you think.

We remind you that this questionnaire is strictly anonymous and confidential.

**1/Demography:**

**Patient number: ____**

**First letter name: ____**

**First letter first name: ____**

**How old are you? : ____ years**

**Are you :** ☐ **Male** ☐ **Female** ☐ **Transgender**

**Where did you born (country of birth)?** : _______

**What is your nationality?** : _______

**If you are not French, since when have you been in France?** : ____ years

2/ Life style :

- What is your marital situation?

☐single ☐ PACS ☐ Married ☐ Divorce ☐ Separate ☐ widow(er)

- Do you live?  ☐ Alone ☐ As a couple ☐ With family
- **What type of accommodation do you have**? ☐lease ☐ owner ☐ staying with friends  ☐ Social center
- **Do you have children**? ☐ Yes ☐ No

If yes, how many? : ____ How many dependent child(ren)? :____

- Do you work? ☐ yes ☐ no

If yes, ☐ full-time ☐ part-time

- What is your profession? : _______
- How long do you estimate your travel time between your home and your office? : ____ minutes
- Do you travel? ☐ Once or twice a year ☐ >2 times a year ☐ Never

☐ Work ☐ Leisure ☐ Return “home”

**3/ Habits**

- **Tobacco smoker?**

☐ yes ☐ no ☐ Former smoker

If yes, how many cigarettes/day: ____________

- **Alcohol consumption?**

☐ yes ☐ no ☐ Former drinker

**If yes, how many glasses do you drink per day?**

☐ Occasionally ☐ 1 to 2 glasses/day ☐ 3 to 5 glasses/day ☐ > 5 glasses/day

- Do you take «recreational drugs»?  ☐ yes ☐ no

**If yes, which one(s)**? : ____________________

**If yes, how often**?

☐ 1time/week ☐ 2 to 3 times/month ☐ 1 time/6 months

**Have you ever used injection drugs?**  ☐ yes ☐ no

**Are you involved in an association fighting against HIV/AIDS ?** ☐ yes ☐no

**4/ Medical history :**

- **Do you have high blood pressure?** ☐ yes ☐ no Treated ☐ yes ☐ no
- **Are you diabetic?** ☐ yes ☐ no Treated ☐ yes ☐ no
- **Do you have cholesterol?**  ☐ yes ☐ no Treated ☐ yes ☐ no
- **Do you have a history of psychiatric disorder or depression?** ☐ yes ☐ no
- **Do you have a psychiatrist/psychologist for your follow-up ?** ☐ yes ☐ no
- **Do you have a treatment if depression?** ☐ yes ☐ no
- **Do you have any other medical history**?  :

☐ Yes ☐ no ; If yes, which one(s) ? : ________

- **For women: do you have contraception?**

☐ Yes ☐ no If yes, which one? *___________*

5/ Regarding your HIV-infection:

- Since when have you been infected with HIV ? : _______________
- How did you get HIV-infection ?

**☐** Injection of drugs **☐** homo/bisexual intercourse

**☐** Heterosexual intercourse ☐soiled equipment

- Are your partner(s) aware of your HIV-infection? ☐ yes ☐ no
- Are your family aware of your HIV-infection? ☐ yes ☐ no
- Are your friends aware of your HIV-infection? ☐ yes ☐ no
- Are your colleagues aware of your HIV-infection? ☐ yes ☐ no
- For how long have you been HIV-treated?

☐ <1 year ☐ 1-5 years ☐ 5-10 years ☐ > 10 years

- Are you taking ART treatment? ☐ once/day ☐ twice/day ☐ >twice/day
- When do you take these ART treatments?

☐ with a meal ☐ while brushing my teeth ☐ when I think about it

- Are you taking any other medications?

☐ yes ☐ no If yes, which ones? : ___________

Frequency: ☐ Once/day ☐ Twice/day ☐ >2 times/day

- Would you say your ART treatment is?

☐ simple ☐ complicated ☐ unmanageable

- Have you had any adverse effects related to your treatments?

☐ Never ☐ yes but minors ☐ yes AND majors

- Are you currently tolerating your ART treatment well? ☐ yes ☐ no
- Do you forget to take your ART treatment? ☐ Never ☐ Sometimes ☐ Regularly
- How often do you go to the hospital to have your HIV infection monitored?

☐ Every 2-3 months ☐ Every 6 months ☐ Every year

6/ **Concerning your experience with ARV treatments**:

- Has your doctor changed your ART treatment for effectiveness problems? ☐ yes ☐ no
- Has your doctor changed your ART treatment for tolerance problems? ☐ yes ☐ no
- Has your doctor changed your ART treatment for adherence problems? ☐ yes ☐ no

7/**Regarding your experience with medication in general**:

• Have you ever had a treatment by injection (= shots)? ☐ yes ☐ no

If yes, for which disease? ☐ Diabetes ☐ Syphilis ☐ Psychiatric illness ☐ HIV

☐ Hepatitis B ☐ Hepatitis C ☐ Other, specify: ______

**Now, suppose that your doctor offers you an ART treatment by injection (= shots) in the department in which you are being followed**

Would you agree to come **every month** for the ART treatment? ☐ yes ☐ no

Would you agree to come **every two months** for the ART treatment? ☐ yes ☐ no

- What would be the advantages for you of such a treatment?

☐ Stop taking treatments every day

☐ Ensure the effectiveness of the treatment for a given period

☐ Be sure not to forget my medication

☐ Being able to hide from surroundings/colleagues that I am taking a treatment

☐ Forget the disease

☐ Not thinking about it daily

☐ Other, specify: _________

- What would be the disadvantages of such treatment for you**?**

☐ Losing my freedom to stop ART treatment whenever I want

☐ Fear of side effects

☐ Fear of injections/pricks

☐ Fear of being taken for a “guinea pig”

☐ Because it wouldn't change my illness

☐ Because I am taking other treatments (for other medical condition)

☐ Other, specify: _________

- **Does the idea of ​​coming to the hospital, having your injections done by a nurse every month, seem to you?**

☐ Beneficial because I will have the impression that I am followed better because more often

☐ I don't like the idea of ​​coming to the hospital more often

☐ I don't care

- **Does the idea of ​​coming to the hospital, having your injections done by a nurse every two months seem to you?**

☐ Beneficial because I will have the impression that I am followed better because more often

☐ I don't like the idea of ​​coming to the hospital more often

☐ I don't care

**Now, suppose that your doctor offers you this treatment by injection, how would you like to integrate it into your ART treatments?**

☐ I do not want to change my treatment

☐ The idea of ​​having an injectable treatment suits me and I do not mind coming to the hospital more often

☐ The idea of ​​having an injectable treatment suits me but it bothers me to come to the hospital more often

☐ The idea of ​​having an injection treatment suits me but I am afraid of the side effects

☐ I would like to be able to switch from my current treatment to this injection treatment at certain times in my life (work, leisure, return home)

What would those moments be?

☐ Holidays ☐ Business trips ☐ Homecoming/events

**If your doctor suggested that you’ll take part in a therapeutic trial evaluating the efficacy of an injectable treatment, what option(s) would be right for you?**

☐ An injection treatment **every month** instead of my current ART treatment

☐ An injection treatment **every month** instead of my current ART treatment, but only at certain times then I will go back to my previous ART treatment

☐ An injection treatment **every two months** instead of my current ART treatment

☐ A treatment by injection **every two months** instead of my current ART treatment but only at certain times, then I will go back to my previous ART treatment

☐ I don't mind coming to have them done at the hospital

☐ It bothers me to come and have them done at the hospital, and I could consider such a treatment if I could do it by myself

☐ I would not wish to participate in such a trial
