# Supplementary material for: Injectable long acting antiretroviral for HIV treatment and prevention: perspectives of potential users
Source: BMC Infect Dis. 2023 Feb 17;23:98. doi: 10.1186/s12879-023-08071-9 (PMC9936705; doi:10.1186/s12879-023-08071-9)
Supplement: Supplementary file 2 — Additional file 2. « Long acting injectable treatment acceptability» PrEP users survey: A survey was built in French by a team of doctors and anthropologists for PrEP users and translate secondarily for publication purpose. Each PrEP user participant gave written informed consent in order to respond to the self-administrated questionnaire. Data collected included demographic parameters, lifestyle, habits, medical history, PrEP history, experiences with medication in particular with PrEP. Perceived advantages and disadvantages of LAA for PrEP. [file 12879_2023_8071_MOESM2_ESM.docx]

**Additional file 2: « Long acting injectable treatment acceptability »**

**PreP user survey**

**Notice :**

We are carrying out a survey on HIV treatment by injection and would like to hear your opinion. As you know, the HIV/AIDS epidemic is a public health problem. If we want to improve the prevention of this disease and in particular HIV-prevention treatments, it is important to collect as much information as possible from concerned individuals. It is therefore very important that your point of view is accurately taken into account.

For this, we would like you to answer an anonymous questionnaire lasting about 10/15 minutes. Thank you very much for your participation in this research and for the time you are willing to devote to it. Your answers will be a very valuable contribution to the research.

For most questions, please give the answer closest to what you think.

We remind you that this questionnaire is strictly anonymous and confidential.

**1/Demography**

**PrEP user Nadis :**

**First letter name:____**

**First letter first name : ____**

**How old are you? ____ years**

**Are you :** ☐ **Male** ☐ **Female** ☐ **Transgender**

**Where did you born (country of birth)?** : ________________

**What is your nationality?** : ______________________

**If you are not French, since when have you been in France?** ____ years

2/ Life style :

- What is your marital situation?

☐single ☐ PACS ☐ Married ☐ Divorce ☐ Separate ☐ widow(er)

- Do you live?  ☐ Alone ☐ As a couple ☐ With family
- **What type of accommodation do you have**? ☐Lease ☐ Owner ☐ Staying with friends  ☐ Social center
- **Do you have children**?  ☐ Yes ☐ No

If yes, how many? : ____ How many dependent child(ren)? : ____

- Do you work? ☐ yes ☐ no

If yes: ☐ full-time ☐ part-time

- What is your profession ? : _________________
- How long do you estimate your travel time between your home and your office? : ____ minutes
- Do you travel? ☐ Once or twice a year ☐ >2 times a year ☐ never
- **Why are you travelling?**

☐ Work ☐ Leisure ☐ Return home

**3/ Habits**

- **Tobacco smoker?**

☐ yes ☐ no ☐ Former smoker

If yes, how many cigarettes/day: ____________

- **Alcohol consumption?**

☐ yes ☐ no ☐ Former drinker

**If yes, how many glasses do you drink per day?**

☐ Occasionally ☐ 1 to 2 glasses/day ☐ 3 to 5 glasses/day ☐ > 5 glasses/day

- Do you take «recreational drugs»?  ☐ yes ☐ no

**If yes, which one(s)** ? : _______

**If yes, how often** ?

☐ 1time/week ☐ 2 to 3 times/month ☐1 time/6 months

**Have you ever used injection drugs?**  ☐ yes ☐ no

**Do you SLAM ?**  ☐ yes ☐ no

**Are you involved in an association fighting against AIDS ?** ☐ yes ☐no

**4/ Medical history :**

- **Do you have high blood pressure?** ☐ yes ☐ no Treated ☐ yes ☐ no
- **Are you diabetic?** ☐ yes ☐ no Treated ☐ yes ☐ no
- **Do you have cholesterol?**  ☐ yes ☐ no Treated ☐ yes ☐ no
- **Do you have a history of psychiatric disorder or depression?** ☐ yes ☐ no
- **Do you have a psychiatrist/psychologist for your follow-up?** ☐ yes ☐ no
- **Do you have a treatment if depression?** ☐ yes ☐ no
- **Do you have any other medical history**?  :

☐ yes ☐ no ; If yes, which one(s) ? : ________

- **For women : do you have contraception ?**

☐ yes ☐ no If yes, which one?*: ___________*

5/ Regarding PreP :

- How many sex partners have you had in the past 3 months? _____________
- When did you start PrEP? ___/____ month/ year
- Does your partner(s) know that you are taking PrEP? ☐ yes ☐ no
- Does your family know that you are taking PrEP? ☐ yes ☐ no
- Do your friends know that you are taking PrEP? ☐ yes ☐ no
- Are your colleagues know that you are taking PrEP? ☐ yes ☐ no
- When do you take your PrEP?

☐ with a meal ☐ while brushing my teeth ☐ when I think about it

- Are you taking any other medications?

☐ yes ☐ no; If yes, which ones ? : _______________

If yes Frequency: ☐ Once/d ☐ Twice/d ☐ >2 times/day

- Would you say your PrEP is? ☐ simple ☐ complicated ☐ unmanageable
- Have you had any adverse effects related to your PrEP?

☐ Never ☐ yes but minors ☐ yes AND majors

- Are you currently tolerating your PrEP well? ☐ yes ☐ no
- Do you forget to take your PrEP? ☐Never ☐Sometimes ☐Regularly

**6/ Regarding your experience with medication in general**:

• Have you ever had a treatment by injection (= shots)? ☐ yes ☐ no

If yes, for which disease? ☐ Diabetes ☐ Syphilis ☐ Psychiatric illness ☐ HIV

☐ Hepatitis B ☐ Hepatitis C ☐ Other, specify: ______

**Now, suppose that your doctor offers you PrEP by injection (= shots) in the department in which you are being followed**

Would you agree to come **every month** for PrEP? ☐ yes ☐ no

Would you agree to come **every two months** for PrEP? ☐ yes ☐ no

- What would be the advantages for you of such PrEP?

☐ Stop taking PrEP every day

☐ Ensure the effectiveness of the PrEP for a given period

☐ Be sure not to forget my medication

☐ Being able to hide from surroundings/ colleagues that I am taking PrEP

☐ Don’t think about it daily

- What would be the disadvantages of such PrEP for you**?**

☐ Losing my freedom to stop PrEP whenever I want

☐ Fear of side effects

☐ Fear of injections/pricks

☐ Fear of being taken for a “guinea pig”

☐ Because it wouldn't change anything

☐ Because I am taking other treatments

- **Does the idea of ​​coming to the hospital, having your PrEP injection done by a nurse every month, seem to you?**

☐ Beneficial because I will have the impression that I am followed better because more often

☐ I don't like the idea of ​​coming to the hospital more often

☐ I don't care

**Now, suppose that your doctor offers you a PrEP by injection, how would you like to integrate it into your PrEP?**

☐ I do not want to change my PrEP

☐ The idea of ​​having an injectable PrEP suits me and I do not mind coming to the hospital more often

☐ The idea of ​​having an injectable PrEP suits me but it bothers me to come to the hospital more often

☐ The idea of ​​having an injection suits me but I am afraid of the side effects

☐ I would like to be able to switch from my current PrEP to this injection treatment at certain times in my life (work, leisure, return home)

What would those moments be?

☐ Holidays ☐ Business trips ☐ Homecoming/events

**If your doctor suggested that you’ll take part in a therapeutic trial evaluating the efficacy of an injectable PrEP, what option(s) would be right for you?**

☐ A PrEP by injection **every month** instead of my current PrEP

☐ A PrEP by injection **every month** instead of my current PrEP, but only at certain times then I will go back to my previous PrEP

☐ A PrEP by injection **every two months** instead of my current treatment

☐ A PrEP by injection **every two months** instead of my current PrEP but only at certain times, then I will go back to my previous PrEP

☐ I don't mind coming to have them done at the hospital

☐ It bothers me to come and have them done at the hospital, and I could consider such PrEP if I could do it by myself

☐ I would not wish to participate in such a trial
