# Supplementary material for: Injectable long acting antiretroviral for HIV treatment and prevention: perspectives of potential users
Source: BMC Infect Dis. 2023 Feb 17;23:98. doi: 10.1186/s12879-023-08071-9 (PMC9936705; doi:10.1186/s12879-023-08071-9)
Supplement: Supplementary file 3 — Additional file 3. « Long acting injectable treatment acceptability» Collected data for PWH by doctors: During the study, data were collected for each participant responding to the self-questionnaire by doctors in the medical file including demographic parameters, habitus, history of HIV (immuno-virologic parameters, ART treatment history, HIV-exposure, ART exposure), comorbidities (HBV, HCV, diabetes, hypertension, dyslipidemia). [file 12879_2023_8071_MOESM3_ESM.docx]

**Additional file 3: “Long acting injectable treatment acceptability”**

**Collected data for PWH by doctors**

**Patient number : _________**

Gender:  Male  Female  Trangender

Date of birth: __/__/____

Native country:

In France since : __/__/____

Weight: ____ kg

Height: ______m

Education level : primary  college  university

**Habitus:**

Tabacco smoking:  never  current  past; if yes, number pack(s)/year : __________

Alcohol consumption: never below 3 glasses/day 3-5 glasses/day  > 5 glasses/day past

Illicit drugs consumption:  never  occasional  regular ( > 3 times/month)

Physical activity: no moderate  ≥3 times/week

For women:

Contraception : :  yes no if yes, which one  ? ______________________

Menopause:  yes no

**History of HIV**

Mode of HIV-transmission: UIVD MSM Heterosexual

CDC Stage:  A  B  C

CD4 nadir: /mm^3^ ( __/__/____)

Curent CD4 : /mm^3^ ( __/__/____)

Curent HIV-RNA : _______ copies/ml ( __/__/____)

**ART exposure:**

Date of first ART initiation: __/__/____

1 st line : 1 NRTI 2 NRTI 2NRTI + NNRTI NRTI+PI/r  2 NRTI+ II other

start date __/____ end __/____

2nd line: 1 NRTI 2 NRTI 2NRTI + NNRTI NRTI+PI/r  2 NRTI+ II  other

start date __/____ end __/____

3 rd line: 1 NRTI 2 NRTI 2NRTI + NNRTI NRTI+PI/r  2 NRTI+ II  other

start date __/____ end __/____

4 th line: 1 NRTI 2 NRTI 2NRTI + NNRTI NRTI+PI/r  2 NRTI+ II  other

start date __/____ end __/____

5 th line: 1 NRTI 2 NRTI 2NRTI + NNRTI NRTI+PI/r  2 NRTI+ II  other

start date __/____ end __/____

6 th line: 1 NRTI 2 NRTI 2NRTI + NNRTI NRTI+PI/r  2 NRTI+ II  other

start date __/____ end __/____

7 th line: 1 NRTI 2 NRTI 2NRTI + NNRTI NRTI+PI/r  2 NRTI+ II  other

start date __/____ end __/____

8 th line: 1 NRTI 2 NRTI 2NRTI + NNRTI NRTI+PI/r  2 NRTI+ II  other

start date __/____ end __/____

9 th line: 1 NRTI 2 NRTI 2NRTI + NNRTI NRTI+PI/r  2 NRTI+ II  other

start date __/____ end __/____

10 th line: 1 NRTI 2 NRTI 2NRTI + NNRTI NRTI+PI/r  2 NRTI+ II  other

start date __/____ end __/____

ART exposure : _____ years

Current ART treatment : ______________________ (start date: __/__/_____)

COMORBIDITIES:

HBV coinfection : yes no

HCV coinfection:  yes no

Dyslipidemia :  yes no Treated  yes no

Diabetes :  yes no Treated :  yes no

High blood pressure :  yes no Treated  yes no
