# Supplementary material for: Injectable long acting antiretroviral for HIV treatment and prevention: perspectives of potential users
Source: BMC Infect Dis. 2023 Feb 17;23:98. doi: 10.1186/s12879-023-08071-9 (PMC9936705; doi:10.1186/s12879-023-08071-9)
Supplement: Supplementary file 4 — Additional file 4. Table S1: Patients characteristics associated with acceptance among PWH currently under single tablet regimen (STR), (N=65): A supplementary analyze was done among PWH treated by STR to compare PWH who would accept to PWH who not accept injections. [file 12879_2023_8071_MOESM4_ESM.docx]

**Additional file 4 : Table S1: Patients characteristics associated with acceptance among PWH currently under a single tablet regimen (STR) (N=65).** *P*-values were obtained by Wilcoxon or Fisher’s exact tests.

| **Variable** | **Would accept injections**  **(N=47)** | **Would not accept injections**  **(N=18)** | **P** |
| --- | --- | --- | --- |
| Median age (IQR), years | 46 (41 - 55) | 51 (47 - 54) | 0.30 |
| Sex, n (%) | 43 (91) | 12 (67) | 0.022 |
| Mode of transmission, n (%) |  |  | 0.035 |
| MSM | 31 (66) | 6 (33) |  |
| Heterosexual | 8 (17) | 8 (44) |  |
| Other* | 8 (17) | 4 (22) |  |
| Family life: single, n (%) | 31 (66) | 8 (44) | 0.16 |
| Has children, n (%) | 15 (32) | 11 (61) | 0.047 |
| Current worker, n (%) | 40 (85) | 12 (67) | 0.16 |
| Travels, n (%) |  |  | 0.85 |
| 1-2/year | 24 (51) | 10 (56) |  |
| > 2/year | 17 (36) | 7 (39) |  |
| Never | 6 (13) | 1 (6) |  |
| Current smokers, n (%) | 10 (21) | 7 (39) | 0.21 |
| Alcohol consumption: yes, n (%) | 39 (83) | 12 (67) | 0.18 |
| Drugs consumption: yes n (%) | 11 (23) | 2 (11) | 0.32 |
| IV drugs consumption: yes n (%) | 6 (13) | 1 (6) | 0.66 |
| CDC stage, n (%) |  |  |  |
| C | 11 (15) | 4 (15) | >0.99 |
| Antiviral treatment duration, n (%) |  |  | 0.22** |
| < 1 y | 4 (9) | 0 (0) |  |
| 1-5 y | 12 (26) | 3 (17) |  |
| 5-10 y | 6 (13) | 4 (22) |  |
| > 10 y | 25 (53) | 11 (61) |  |
| Median CD4 (IQR), cells/µl | 746 (521 - 1002) | 705 (528 - 805) | 0.56 |
| Median CD4 nadir (IQR), cells/µl | 338 (185 - 446) | 292 (150 - 439) | 0.34 |
| Hypertension, n (%) | 13 (28) | 6 (33) | 0.76 |
| Diabetes, n (%) | 3 (6) | 0 (0) | 0.55 |
| Hypercholesterolemia, n (%) | 10 (21) | 9 (50) | 0.033 |
| Psychiatric disorder, n (%) | 9 (19) | 1 (6) | 0.26 |
| Antidepressant, n (%) | 4 (9) | 1 (6) | >0.99 |
| Taking any non-HIV treatment, n (%) | 20 (43) | 8 (44) | >0.99 |
| Partner aware of treatment, n (%) | 39 (83) | 13 (72) | 0.49 |
| Family aware of treatment, n (%) | 21 (45) | 7 (39) | 0.78 |
| Friends aware of treatment, n (%) | 23 (49) | 4 (22) | 0.090 |
| Colleagues aware of treatment, n (%) | 8 (17) | 1 (6) | 0.42 |
| Never experienced AEs, n (%) | 28 (60) | 14 (82) | 0.14 |
| Never forgets treatment, n (%) | 32 (68) | 12 (67) | >0.99 |
